# Applied Trace Alkali Metal Elements for Semiconductor Property Modulation of Perovskite Thin Films

**DOI:** 10.3390/molecules24224039

**Published:** 2019-11-07

**Authors:** Chuangchuang Chang, Xiaoping Zou, Jin Cheng, Tao Ling, Yujun Yao, Dan Chen

**Affiliations:** 1 Beijing Advanced Innovation Center for Materials Genome Engineering, Research Center for Sensor Technology, Beijing Key Laboratory for Sensor, MOE Key Laboratory for Modern Measurement and Control Technology, School of Applied Sciences, Beijing Information Science and Technology University, Jianxiangqiao Campus, Beijing 100101, China; changcc037@gmail.com (C.C.); chengjin@bistu.edu.cn (J.C.); taoling8102@gmail.com (T.L.); yyj10zy@gmail.com (Y.Y.); 2 State Key Laboratory on Integrated Optoelectronics, Institute of Semiconductors, Center of Materials Science and Optoelectronics Engineering, University of Chinese Academy of Sciences, Chinese Academy of Sciences, Beijing 100864, China; danchen630@gmail.com

**Keywords:** alkali metal, perovskite solar cells, N-type, P-type, homojunction, doping, films

## Abstract

With the rapid consumption of energy, clean solar energy has become a key study and development subject, especially the when new renewable energy perovskite solar cells (PSCs) are involved. The doping method is a common means to modulate the properties of perovskite film. The main work of this paper is to incorporate trace amounts of alkali metal elements into the perovskite layer and observe the effects on the properties of the perovskite device and the majority carrier type of the perovskite film. Comparative analysis was performed by doping with Na^+^, K^+^, and Rb^+^ or using undoped devices in the perovskite layer. The results show that the incorporation of alkali metal ions into the perovskite layer has an important effect on the majority carrier type of the perovskite film. The majority carrier type of the undoped perovskite layer is N-type, and the majority carrier type of the perovskite layer doped with the alkali metal element is P-type. The carrier concentration of perovskite films is increased by at least two orders of magnitude after doping. That is to say, we can control the majority of the carrier type of the perovskite layer by controlling the doping subjectively. This will provide strong support for the development of future homojunction perovskite solar cells. This is of great help to improve the performance of PSC devices.

## 1. Introduction

The astonishing development of the economy has imposed challenges for the energy industry and made it indispensable [1]. However, fossil energy is not renewable and causes serious environmental pollution. In contrast, solar energy is an inexhaustible source of clean energy. At present, the mature devices on the market are mainly silicon solar cells. Thin film solar cells are developing rapidly, especially perovskite solar cells (PSCs). The device efficiency certified by the US National Renewable Energy Laboratory recently reached 25.2% [2]. Perovskite materials have become the leader in new solar materials due to their many excellent material properties, such as suitable band gap, long carrier diffusion length, long minority carrier lifetime, low cost, and simple preparation [3,4,5,6,7]. However, there are still some problems in perovskite materials, such as efficiency, toxicity, marketization, and stability [8,9]. More research and exploration are still needed. In order to control the properties of perovskite thin films, common means include doping technology, the film forming process, environmental control, and so on. Most of the previous studies used doping to enhance the planar heterojunction [10,11] to improve device performance, essentially reducing the carrier recombination loss of the carrier to reduce the defects of the perovskite film [12,13,14].

Huang et al. reviewed the effects of metal ions on the performance of PSC devices [15]. For example, Na^+^ doping can reduce nonradiative and recombination rate, improve grain size, and reduce grain boundary and trap density [10,16,17]. K^+^ doping can improve grain size and reduce grain boundary and trap density [10,17]. Rb^+^ doping leads to passivation of grain boundaries [18]. Although there have been many studies on the influence of alkali metal doping on PSC devices, the modulation of perovskite semiconductor types by alkali metal doping is still rare. The use of metal nano-ions can increase light trapping ability and expand the range of light absorption [19]. Although some metal ions have already proved effective in modulating bandgaps through alloying, their ability to control crystallization, carrier concentration, and emissive effects still require significant improvements in fundamental understanding [15].

As early as 2014, the research group of Tingting Shi calculated the doping method of perovskite solar cells based on density functional theory, and predicted that P-type perovskite solar cells could be prepared by incorporating alkali metal elements into perovskite materials under certain conditions [20]. In 2016, Wang et al. studied improving crystal quality by doping Al^3+^ to reduce microstrain in polycrystalline films [21]. In 2017, Liu et al. studied one-step doping with Na^+^ and K^+^ to reduce trap density to improve device performance [17]. In 2018, our research group (Bai et al.) found that the incorporation of Rb^+^ into the perovskite-absorbing material by a one-step process could change the semiconductor type and carrier concentration of perovskite [22]. Theoretical simulations and experimental results show that perovskites can be prepared into P-type or N-type by specific means [23,24,25,26,27]. Moreover, concerning modulating the carriers type of behavior, it is also relevant to look at the internal charge anomaly distribution of PSCs [28]. These studies provide a theoretical basis for the extrinsic doping of perovskite to change the majority of carrier types, contributing to solving challenges impeding the development of homojunction in perovskite. 

Here we assume that if the majority carrier type of the perovskite film can be controlled subjectively, the composite perovskite layer with homojunction can be prepared step by step. The homojunction inside the perovskite film can better improve the separation and migration ability of carriers by forming the built-in electric field [12], thereby reducing the defects of the perovskite film. Huang et al. self-doped the perovskite into P-type or N-type by controlling the ratio of the two precursors of perovskite [23]. Li et al. prepared P-type or N-type perovskite [12] by controlling the preparation process and growth conditions. In this paper we intend to further systematically introduce the effect of external doping of alkali metal elements on the majority carrier type of perovskite.

In this paper, we prepared perovskite solar cells by two-step method and doped trace alkali metal elements in PbI_2_ precursor solution. We prepared four different groups using Na^+^-, K^+^-, and Rb^+^-doped as well as undoped samples. We used different alkali metal elements to study the extrinsic doping of the perovskite layer, and experimental data showing that CH_3_NH_3_PbI_3_ (MAPbI_3_) can be used for extrinsic doping from the N-type to P-type are provided. This revealed that the physical properties of perovskite thin films can be modulated by controlling precursor solution compositions and doping craft, leading to the change in the majority carrier type and demonstrating a promising platform for opening new horizons in homojunction PSCs.

## 2. Materials and Experimental Methods

### 2.1. Materials

The conductive glass is a transparent conductive SnO_2_ glass doped with fluorine (FTO) as the substrate of the thin-film solar cell. It was purchased from Shanghai MaterWin New Materials Co., Ltd. (Shanghai, China, 7–8 Ω/square, 2.2 mm in thickness, 1.5 × 1.5 cm^2^ in specification). Dimethyl sulfoxide (DMSO) and N, N-dimethyl formamide (DMF) were purchased from Alfa Aesar (China) Co., Ltd. (Shanghai, China). Acid titanium dioxide solution (bl-TiO_2_, product code MTW-CL-H-002, commodity name HH-TiOx, colorless and transparent in appearance, 99.98% purity), 18NR-T TiO_2_ (mp-TiO_2_, product code MTW-CL-H-001, commodity name 18NR-T TiO_2_, beige paste in appearance, solid content 4%), isopropanol (IPA, CAS No. 67-63-0, colorless and transparent in appearance, 99.8% purity), and chlorobenzene (CAS No. 108-90-7, colorless and transparent in appearance, 99.8% purity) were purchased from Shanghai MaterWin New Materials Co., Ltd. (Shanghai, China). Spiro-OMeTAD solution (Spiro-OMeTAD, CAS No. 207739-72-8, yellow powder in appearance, purity ≥99.5%), methylammonium iodide (MAI, CAS No. 14965-49-2, white powder in appearance, purity ≥99.5%), PbI_2_ (CAS No. 10101-63-0, yellow crystalline powder in appearance, purity >99.99%), NaI (CAS No. 7790-29-6, white granular, 99.9% in purity), KI (CAS No. 7681-11-0, white granular, purity ≥99.999%), and RbI (CAS No. 7681-82-5, white granular, 99.999% purity) were obtained from Xi’an Polymer Light Technology Corp. (Xi’an China). 

### 2.2. Device Fabrication

The overall structure of the device is FTO/bl-TiO_2_/mp-TiO_2_/CH_3_NH_3_PbI_3_/Spiro/FTO(C), from bottom to top. FTO conductive glass is a photoanode material for solar cell devices, which must be cleaned before use. After cutting the glass, ultrasonic cleaning of FTO was carried out successively with mixed solution (detergent and deionized water), glass water (acetone: deionized water: 2-propanol = 1:1:1), and alcohol. Washed with deionized water and dry for 30 min, the FTO was ozone-treated with ultraviolet ozone (UVO) for one hour before use. The compact TiO_2_ layer (bl-TiO_2_) was spin-coated with a layer of acidic TiO_x_ solution at a rate of 2000 rpm for 60 s, and then heated to 100 °C for 10 min on a hot plate. Finally in the muffle furnace, 30 min under 500 °C calcination resulted in a smooth TiO_2_-dense layer. The TiO_2_ mesoporous layer (mp-TiO_2_) was spin-coated with a layer of the 18NR-T TiO_2_ slurry solution at 2000 rpm for 30 s, and was afterward heated on the hot plate to 100 °C for 10 min. Finally, in the muffle furnace, 500 °C calcination for 1 h was used to obtain a uniform mesoporous TiO_2_ layer.

The perovskite layer was prepared using two solution deposition methods. On weighing 0.5993 g PbI_2_ (1.3 mol/L) with an electronic balance and baking it on a hot plate at 70 °C for 30 min, PbI_2_ changed from light yellow to orange. Then, the precursor PbI_2_ was dissolved using dimethyl sulfoxide (DMSO) and N, N-dimethylformamide (DMF) as two kinds of solvents (volume ratio of 0.05:0.95) in the mixed solution. Four PbI_2_ precursor solutions were prepared in this experiment, which were undoped, doped with a concentration of 0.04 M/L NaI, doped concentration of 0.04 M/L KI, and doped concentration of 0.04 M/L RbI. Then, 0.06 g of NaI, 0.0664 g of KI, and 0.0848 g of RbI were respectively incorporated into three bottles of the PbI_2_ precursor solution. They were placed in an ultrasonic device for sonication until the solute was completely dissolved; then the solution was filtered and the PbI_2_ solution was ready. MAI (0.07 g) was weighed with an electronic balance, and 1 mL of isopropanol (solvent) was added. This was placed in an ultrasonic device for sonication until the solute was completely dissolved, the solution was filtered, and then the MAI solution was ready. The first step in the two-step process was to spin coat the PbI_2_ solution and spin it on the mesoporous film at 1500 rpm for 30 s. The second step was to spin-coat the MAI solution on the newly formed PbI_2_ film at 1500 rpm for 30 s, and then place it on a hot plate at 150 °C for 15 min. The perovskite film was then obtained. An appropriate amount of Spiro spin coating was applied to the perovskite film. The hole transport layer was obtained by rotating it for 30 s at the rate of 3000 rpm. Finally, the counter electrode was cleaned with FTO as the substrate, and the smoke particles generated by candle combustion formed the carbon film. The prepared carbon film was aligned on the top layer of the prepared device. A small clip was used to clamp the sides for easy packaging. 

### 2.3. Characterization

The morphology details of perovskite films were measured by scanning electron microscope (SEM) (SIGMA, Zeiss, Jena, Germany). Energy dispersive X-ray spectroscopy (EDS) was used for testing and analyzing the content of chemical constituents in perovskite films. X-ray diffraction (XRD) data from samples of perovskite thin films substrates were collected using an X-ray diffractometer (D8 Focus, Bruker, Dresden, Germany). The four different sets of samples were analyzed using an ultraviolet (UV) visible absorption spectrometer (Avantes, Apeldoom, the Netherlands) and the Photoluminescence (PL) Spectroscopy data were obtained by a LabRAW HR800 PL testing system (HORIBA JObin Yvon, Paris, France). Hall effect data were measured by the Hall Effect Measurement System HL5500PC (QUATEK, Shanghai, China). The photocurrent density-voltage (J-V) characteristics were measured under simulated standard air quality daylight (AM 1.5, 100 mW/cm2) with a solar simulator (Sol 3A, Oriel, New Port, RI, USA).

The Hall effect test described in this paper used a perovskite film prepared on a glass substrate. In other tests, perovskite film refers to the film prepared on carrier transport layer. All the tests contain substrates. The perovskite layer described refers to a single layer of perovskite, and the test results do not include other layer.

## 3. Results and Discussion

**Figure 1:**
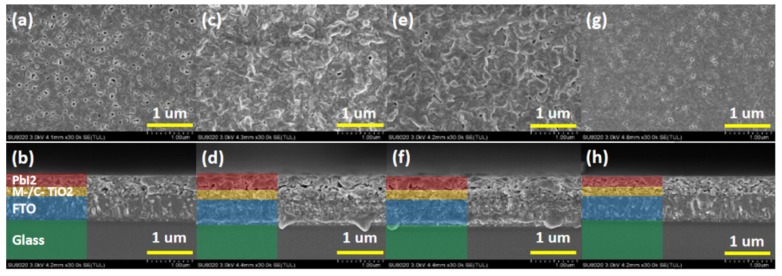
Top view and cross-sectional view of the alkali metal-doped PbI_2_ film for (**a**,**b**) PbI_2_, (**c**,**d**) PbI_2_ + 0.04NaI, (**e**,**f**) PbI_2_ + 0.04KI, (**g**,**h**) PbI_2_ + 0.04RbI. The PbI_2_ films that are undoped, have doped concentration of 0.04 M/L NaI, doped concentration of 0.04 M/L KI, and doped concentration of 0.04 M/L RbI are referred to as PbI_2_, PbI_2_ + 0.04NaI, PbI_2_ + 0.04KI, and PbI_2_ + 0.04RbI, respectively.

The PbI_2_ films that are undoped, have a doped concentration of 0.04 M/L NaI, doped concentration of 0.04 M/L KI, and doped concentration of 0.04 M/L RbI were named as PbI_2_, PbI_2_ + 0.04NaI, PbI_2_ + 0.04KI, and PbI_2_ + 0.04RbI, respectively. All of the PbI_2_ films in the paper were deposited on the TiO_2_ layer. Figure 1 shows the top view and cross section view of PbI_2_ thin film; (a) and (b) correspond to the top view and cross-sectional view of the undoped PbI_2_ thin film, as a reference sample. Figure 1c,d correspond to the top view and cross-sectional view of PbI_2_ + 0.04NaI thin film, Figure 1e,f correspond to the top view and cross-sectional view of PbI_2_ + 0.04KI thin film, and Figure 1g,h correspond to PbI_2_ + 0.04RbI thin film. Top and cross-sectional views are provided, with magnification of 30,000 times, and the scale line is 1 μm. 

It can be seen from Figure 1 that whether PbI_2_ films are doped or not, they are not very flat and dense, and there are many pinholes in the undoped films. On the one hand, these pinholes reduce the crystallinity of PbI_2_ films. On the other hand, it is beneficial for MAI solution to enter into PbI_2_ film and react with it, thereby facilitating the formation of perovskite film. It can be seen from the cross-sectional view in Figure 1a that there are holes in the undoped PbI_2_ film. From Figure 1c, it can be seen that the surface of the PbI_2_ layer doped with Na^+^ is the roughest, and the hole is also found to be the largest in the cross-sectional view, almost forming an isolation layer. In Figure 1e,f, the surface of the PbI_2_ layer doped with K^+^ is also rough, but the pores and roughness are lower than those doped with Na^+^. It is found in Figure 1g,h that the film of the PbI_2_ layer doped with Rb^+^ is relatively flat and has fewer holes than the surface of the undoped film.

**Figure 2:**
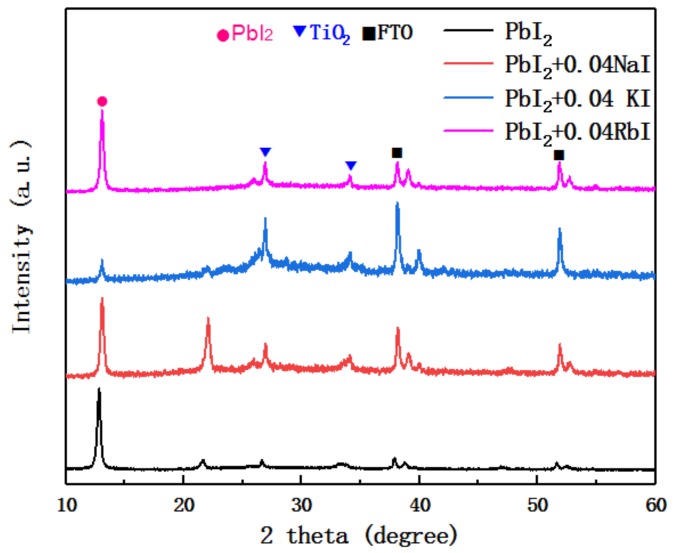
XRD patterns of undoped and alkali metal-doped PbI_2_ films.

Figure 2 shows the XRD patterns of the undoped PbI_2_ film and the PbI_2_ film doped with trace alkali metal elements. The data were measured on a PbI_2_ film deposited on a mesoporous layer of TiO_2_. The peaks in Figure 2 include the TiO_2_ peak and the FTO peak. It can be seen from the figure that the intensity of the first main peak of PbI_2_ (about 12° position) is significantly reduced after doping with alkali metal elements, and has the largest change after incorporation of K^+^. The change after doping with Na^+^ and Rb^+^ is small. This indicates that with the addition of alkali metal elements, the crystallinity of PbI_2_ is affected. In addition, the second main peak of PbI_2_ (about 22° position) was found to increase in strength when Na^+^ was incorporated, and the peak position is shifted to the right. The intensity was almost zero after incorporation of K^+^ and Rb^+^. This indicates that the PbI_2_ crystal type is distorted with the incorporation of an alkali metal element.

**Figure 3:**
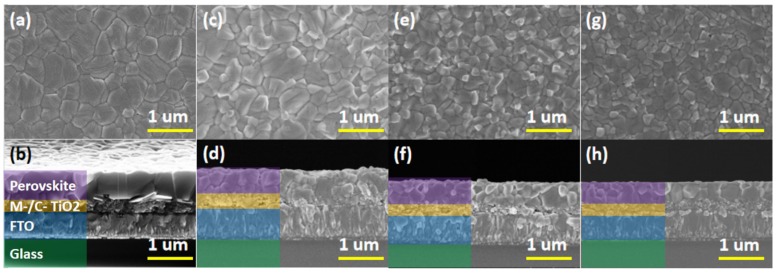
Top view and cross-sectional view of the alkali metal-doped MAPbI_3_ film for (**a**,**b**) MAPbI_3_, (**c**,**d**) MAPbI_3_ + 0.04NaI, (**e**,**f**) MAPbI_3_ + 0.04KI, and (**g**,**h**) MAPbI_3_ + 0.04RbI. The perovskite films that are undoped, have an doped concentration of 0.04 M/L NaI, doped concentration of 0.04 M/L KI, and doped concentration of 0.04 M/L RbI are referred to as MAPbI_3_, MAPbI_3_ + 0.04NaI, MAPbI_3_ + 0.04KI, and MAPbI_3_ + 0.04RbI, respectively.

The perovskite films that are undoped, have an doped concentration of 0.04 M/L NaI, doped concentration of 0.04 M/L KI, and doped concentration of 0.04 M/L RbI were named as MAPbI_3_, MAPbI_3_ + 0.04NaI, MAPbI_3_ + 0.04KI, and MAPbI_3_ + 0.04RbI, respectively. Figure 3 provides a top and cross-sectional view of an alkali metal doped perovskite film. Figure 3a,b correspond to the top view and cross-sectional view of the undoped MAPbI_3_ thin film, as a reference sample. Figure 3c,d correspond to the top and cross-sectional view of MAPbI_3_ + 0.04NaI thin film, Figure 3e,f correspond to the top view and cross-sectional view of MAPbI_3_ + 0.04KI thin film, and Figure 3g,h correspond to MAPbI_3_ + 0.04RbI thin film. Top and cross-sectional views are provided, with magnifications of 30,000 times, and the scale line is 1 μm. 

It can be seen from Figure 3a that the top view of the undoped perovskite film is flat, the perovskite crystal grain is larger, the maximum diameter is about 800 nm, and the grain size is different, but the grain boundaries are obvious. Figure 3b provides a corresponding cross-sectional view of the perovskite layer, the TiO_2_ mesoporous layer, the TiO_2_ dense layer, and FTO from top to bottom. It can be observed that the perovskite grains penetrate the entire perovskite layer, the grain thickness is the thickness of the film, and the thickness is about 680 nm. Figure 3c shows the top view of MAPbI_3_ + 0.04NaI film. Compared with the undoped perovskite film, the grain size of MAPbI_3_ + 0.04NaI film does not increase, the surface flatness is reduced, and no pinholes appear. Figure 3d provides a corresponding cross-sectional view. It can be seen that the vertical grain boundary of the MAPbI_3_ + 0.04NaI film is significantly increased relative to the undoped perovskite film, and the grain no longer runs through the whole perovskite layer. Figure 3e shows the top view of the MAPbI_3_ + 0.04KI film. Compared to the undoped perovskite film, it can be seen that the surface becomes uneven and the grain size becomes smaller. The white part of the figure indicates the protruding part. Figure 3f provides its corresponding cross-sectional view; it can be seen that the surface of the film is high and low, and the flatness is reduced. The grain boundary in the vertical direction of the perovskite film is obviously increased, and more perovskite grains no longer penetrate the entire perovskite layer, but the film is dense and has no pores. Figure 3g shows the top view of the MAPbI_3_ + 0.04M RbI-doped perovskite film. Compared with the undoped perovskite film, the grain size is obviously reduced, and the maximum is about 300 nm. Moreover, a white phase appeared, which indicates that the surface flatness is lowered. From the corresponding cross-sectional view (h), it can be seen that the grain boundary of the perovskite film is significantly increased in the vertical direction, the grain size is small, and no holes are formed.

**Figure 4:**
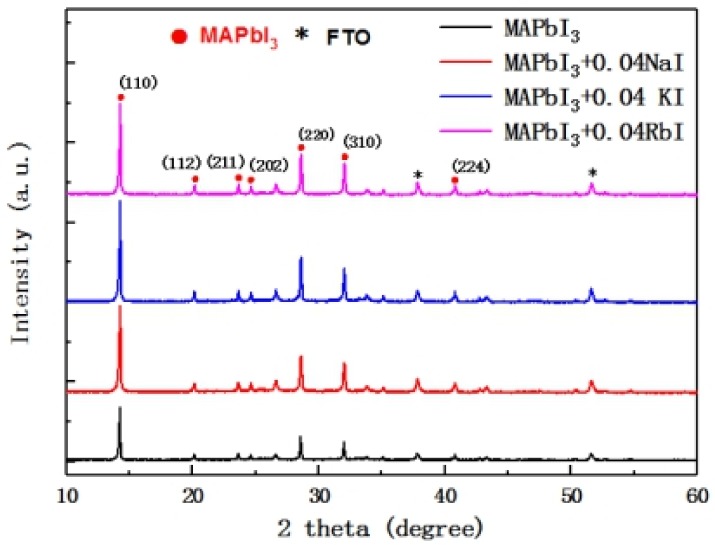
XRD patterns of undoped and alkali metal-doped MAPbI_3_ films.

Figure 4 shows the XRD patterns of the undoped MAPbI_3_ film and the MAPbI_3_ film doped with trace alkali metal elements. It can be seen from the diagram that the intensity of the first main peak of the perovskite (about a 14° position) increases with the incorporation of alkali metal elements. This indicates that the crystallinity of the perovskite increases with the incorporation of alkali metal elements. It is obvious that there is no significant change in the position of all the XRD peaks of the perovskite. That is, no new peak appeared and no peak disappeared after doping, indicating that the crystal structure of the perovskite did not change significantly. 

**Figure 5:**
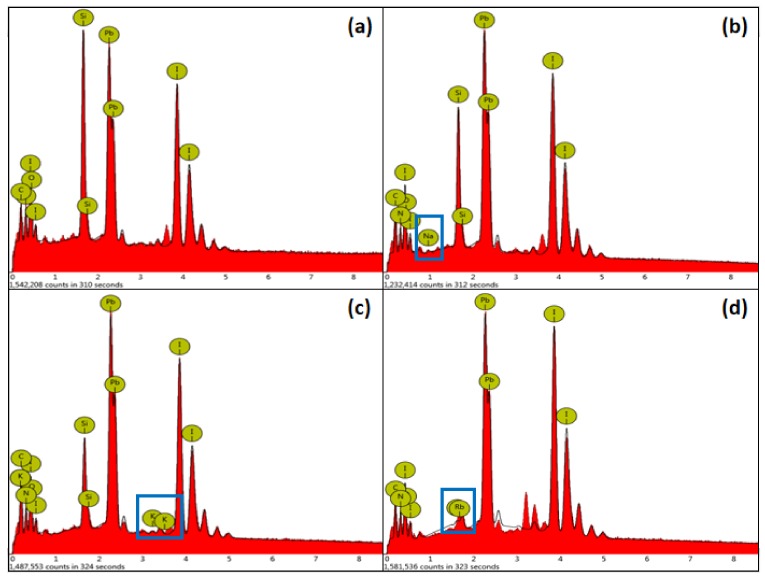
Undoped and alkali metal-doped perovskite film EDS images, (**a**) MAPbI_3_, (**b**) MAPbI_3_ + 0.04NaI, (**c**) MAPbI_3_ + 0.04KI, (**d**) MAPbI_3_ + 0.4RbI.

Figure 5 is an EDS diagram of the alkali metal element-doped perovskite film, the peaks of which are indicated by blue rectangles in the corresponding figures, indicating that the alkali metal elements are successfully incorporated into the perovskite film. Table 1 shows the atomic percentage of EDS of the alkali metal element-doped perovskite film. It can be seen that the atomic percentage of the alkali metal element is shown in the corresponding data, which confirms that the alkali metal element has been incorporated into the perovskite film. Oxygen and silicon may come from the glass substrate.

Table 2 shows the Hall test parameters for the alkali metal element-doped perovskite layer. As can be seen from the table, the undoped perovskite layer has a negative majority carrier concentration and is an N-type semiconductor. After the alkali metal element is doped, the majority carrier concentration of the perovskite layer has a positive value and is a P-type semiconductor. This indicates that the incorporation of the alkali metal element changes the perovskite from an N-type semiconductor to a P-type semiconductor. This is consistent with the results predicted by Shi et al. according to density functional theory [20]. Combined with the above data, it is found that the preparation of the perovskite layer with a trace amount of alkali metal elements can change the majority carrier type of the perovskite layer. That is to say, via controlling the incorporation of alkali metal elements, we can subjectively modulate the majority carrier type of the perovskite film. This lays the foundation for the next study of homojunction perovskite solar cells.

The results of the Hall test on semiconductor types are consistent with the literature [22,29]. The undoped perovskite layer semiconductor type is N-type, the doped semiconductor type is P-type, and the carrier concentration is slightly higher herein [30]. The carrier concentration after doping with metal ions is also increased [31].

**Figure 6:**
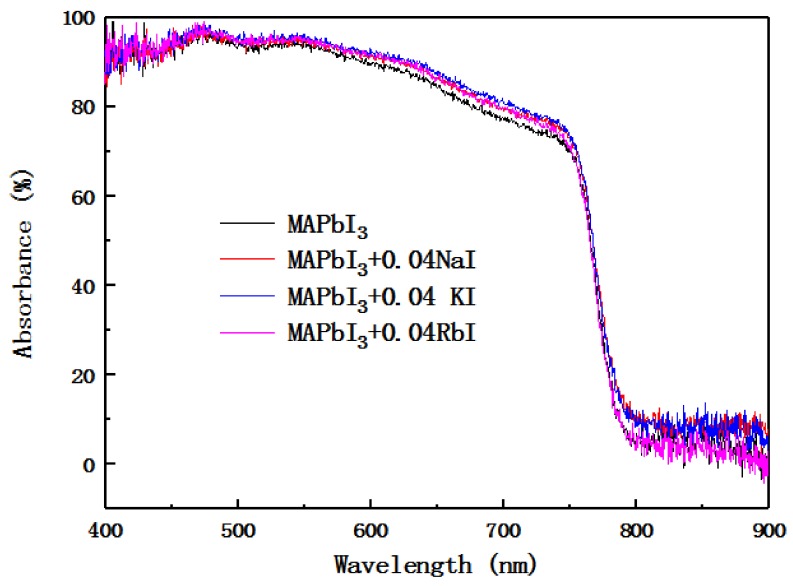
UV-visible absorption spectra of perovskite films with the undoped and the alkali metal-doped layers.

Figure 6 is the UV absorption spectrum of perovskite layer doped with alkali metal elements. It can be seen from the diagram that the UV absorption spectrum intensity of the alkali metal-doped perovskite layer is greater than the undoped perovskite layer UV absorption spectrum intensity in the visible light wavelength range. Doping with Na^+^ and K^+^ causes the absorption band edge to show a red shift, and doping with Rb^+^ causes a slight blue shift in the absorption band edge. 

After calculation, the band gap of the undoped sample is 1.56 eV, the band gap of the Na^+^-doped sample is 1.55 eV, the band gap of the K^+^ -doped sample is 1.54 eV, and the band gap of the Rb^+^-doped sample is 1.57 eV. The incorporation of Na^+^ or K^+^ resulted in a red shift in absorption edge and band gap shrinkage, whereas the incorporation of Rb^+^ caused a blue shift and band gap increase. The identical trend was also found in the photoluminescence spectra, indicating the modification of the band gap by the alkali metal cations. The experimental results are consistent with the literature [32].

**Figure 7:**
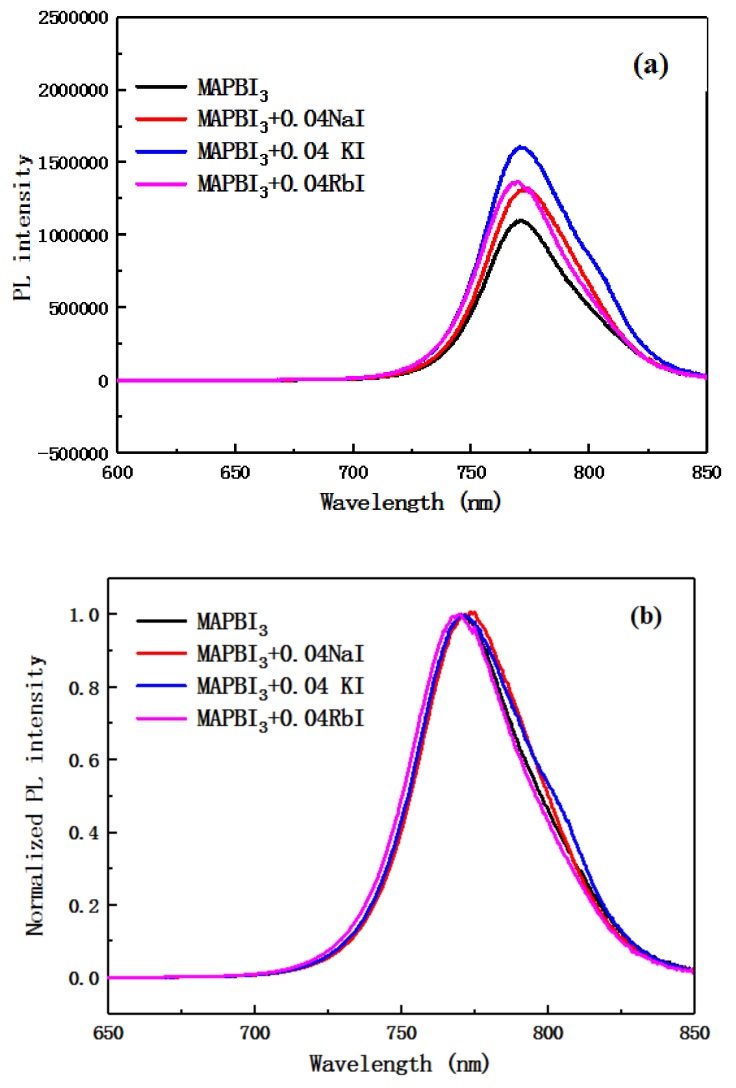
Photoluminescence spectra of perovskite films with the undoped and the alkali metal-doped layers. Unsmoothed (**a**) and smoothed after (**b**).

Figure 7 include the PL spectrum and a normalized PL spectrum of a perovskite layer doped with an alkali metal element. It can be seen from the figure that as the photoluminescence intensity of the alkali metal element incorporated into the perovskite layer increases, the ability of carriers to implant into the TiO_2_ layer is weakened. From the normalized PL spectrum, the emission peaks of the photoluminescence spectra doped with Na^+^ and K^+^ show a red shift, and the doping of Rb^+^ causes a slight blue shift in the emission peak. This is consistent with the display of the UV absorption spectral shift of Figure 6. This also reflects a change in the band gap width of the perovskite after doping, which may change the film type of the perovskite layer.

**Figure 8:**
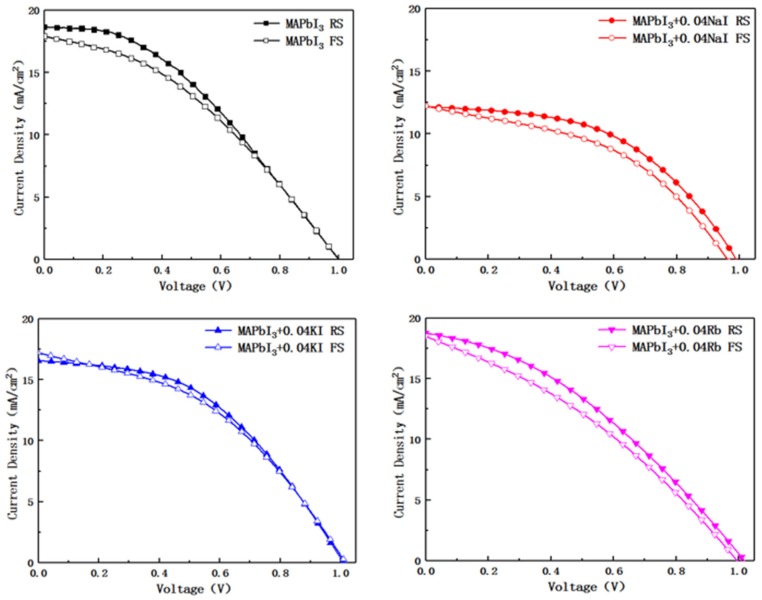
Photocurrent voltage density Reverse Scan and Forward Scan curve of undoped and alkali metal-doped devices.

Figure 8 shows the Reverse Scan (RS) and Forward Scan (FS) J-V curve for four sample perovskite devices. Table 3 shows the photovoltaic parameters of the four sample perovskite devices. After the alkali metal element is doped, the open circuit voltage shows a slight floating, but the open circuit voltage of the device doped with K^+^ is the largest. It can be seen from Figure 8 that the device with K^+^ doping has the lowest hysteresis. This may be the mainly reason for the best performance of K^+^-doped devices. This paper selects typical data from the prepared samples [33]. The spongy carbon is employed as counter electrode in perovskite photovoltaic devices. The preparation of the entire installation is done in the air.

## 4. Conclusions

In this paper, the comparative analysis of undoped and alkali metal-doped perovskite devices was performed. Scanning electron microscopy and XRD patterns were performed on the PbI_2_ film and the perovskite film of the samples. Although the incorporation of alkali metal elements has a great influence on the morphology and crystal structure of the PbI_2_ film, it has little effect on the crystal structure of the perovskite film prepared by the two-step method. The EDS test confirmed that alkali metal elements were successfully incorporated into perovskite layer. The UV-visible absorption spectrum showed that the samples doped with alkali metal elements had higher absorption intensity. The incorporation of Na^+^ or K^+^ resulted in a red shift in absorption edge and band gap shrinkage, whereas the incorporation of Rb^+^ caused a blue shift and band gap increase. It can be seen from the from the photoluminescence spectrum that the undoped sample perovskite layer carrier is more capable of injecting the TiO_2_ layer. Compared to the alkali metal element-doped samples, the undoped perovskite layer has a larger grain size, a more complete grain, and fewer grain boundaries.

The Hall effect test was also carried out in this experiment. The results show that the majority carrier type of the undoped perovskite layer prepared by the two-step method is the N-type, and the majority carrier type of the perovskite layer doped with alkali metal element is the P-type. This is consistent with the results predicted by Shi et al. according to density functional theory [20]. The carrier concentration of perovskite films is increased by at least two orders of magnitude after doping. In summary, we presented a method to modulate majority carrier type from perspective of extrinsic doping, which will contribute to the performance optimization of PSCs. That is to say, we can subjectively control the majority carrier type of perovskite film by controlling the experimental process and doping composition. This experimental conclusion is significant and will lay a solid foundation for the subsequent homojunction perovskite solar cell.

## Figures and Tables

**Table 1:** Undoped and alkali metal-doped perovskite film EDS atomic percentage.

Samples	Na	K	Rb	N	C	I	Pb
MAPbI_3_	0	0	0	27.71	26.77	26.31	19.20
MAPbI_3_ + 0.04NaI	0.36	0	0	20.96	29.03	28.79	20.86
MAPbI_3_ + 0.04 KI	0	0.28	0	20.67	34.90	25.46	18.69
MAPbI_3_ + 0.04RbI	0	0	0.32	21.88	22.98	33.98	20.84

**Table 2:** Hall test parameters of the undoped and alkali metal-doped perovskite layer.

Samples	Rs ^a^ (ohm/sq)	Mob ^b^ (cm^2^/Vs)	N ^c^ (/cm^3^)	Type ^d^
MAPbI_3_	4.673 × 10^9^	494	−5.406 × 10^10^	N
MAPbI_3_ + 0.04NaI	7.242 × 10^7^	435	+3.958 × 10^12^	P
MAPbI_3_ + 0.04 KI	9.57 × 10^8^	56.6	+2.306 × 10^12^	P
MAPbI_3_ + 0.04RbI	2.043 × 10^9^	1.7	+3.591e × 10^13^	P

Notes: a. Surface resistivity; b. Hall mobility; c. Carrier concentrations; d. Majority carrier type.

**Table 3:** The photovoltaic parameters of undoped and alkali metal-doped devices.

Samples	RS/FS	V_oc_^a^ (V)	J_sc_^b^ (mA/cm^2^)	FF ^c^ (%)	PCE ^d^ (%)
MAPbI3	RS	1.00	18.67	38.42	7.17
MAPbI3	FS	1.00	17.94	37.57	6.73
MAPbI3 + 0.04NaI	RS	0.99	12.18	49.32	5.94
MAPbI3 + 0.04NaI	FS	0.96	12.24	44.60	5.24
MAPbI3 + 0.04 KI	RS	1.01	16.58	45.58	7.63
MAPbI3 + 0.04 KI	FS	1.02	17.24	41.97	7.35
MAPbI3 + 0.04RbI	RS	1.02	18.78	35.83	6.85
MAPbI3 + 0.04RbI	FS	0.99	18.53	33.49	6.18

Notes: a. Open-circuit voltage; b. Short-circuit photocurrent density; c. Fill factor; d. Power conversion efficiency.
